# Risk Markers for Not Returning to Work Among Patients with Acquired Brain Injury: A Population-Based Register Study

**DOI:** 10.1007/s10926-019-09833-6

**Published:** 2019-03-04

**Authors:** Marie Matérne, Thomas Strandberg, Lars-Olov Lundqvist

**Affiliations:** 1 University Health Care Research Center, Faculty of Medicine and Health, Örebro University, Örebro, Sweden; 2 School of Law, Psychology and Social Work, Örebro University, Örebro, Sweden; 3 The Swedish Institute for Disability Research, Örebro University, Örebro, Sweden

**Keywords:** Brain Injuries, Return to work, Rehabilitation, vocational, Employment, Registries

## Abstract

*Purpose* The aim of this study is to investigate person-related, injury-related, activity-related and rehabilitation-related risk markers for not return to work among patients with acquired brain injury (ABI). *Methods* Retrospective data from the Quality register, WebRehab Sweden, on an ABI cohort of 2008 patients, was divided into two groups: those who had returned to work (n = 690) and those who had not returned to work (n = 1318) within a year of the injury. *Results* Risk ratio analyses showed that several factors were risk markers for not returning to work: personal factors, including being a woman, being born outside of Sweden, having a low education level, and not having children in the household; injury-related factors, including long hospital stay (over 2 months), aphasia, low motor function, low cognitive function, high pain/discomfort, and high anxiety/depression; activity-related factors, including low function in self-care, inability to perform usual activities, and not having a driver’s license; and rehabilitation-related factors, including being dissatisfied with the rehabilitation process and the attentiveness of the staff having limited influence over the rehabilitation plan, or not having a rehabilitation plan at all. *Conclusion* Several factors in different aspects of life were risk markers for not returning to work among patients with ABI. This suggests that rehabilitation and interventions need to address not only direct injury-related issues, but also person-related, activity-related, and rehabilitation-related factors in order to increase the patient’s opportunities to return to work.

## Introduction

One of the main causes of disability worldwide is acquired brain injury (ABI), which may result from cerebrovascular accidents, infections, toxins, tumors, or trauma to the brain [1–5]. Most people with ABI want to live a normal everyday life after the injury, such as returning to work, earning their subsistence and participating in society [6]. However, for many individuals with ABI it is a challenge to return to work. In Sweden, approximately 26,500 people are affected by stroke annually [7], a further 14,000 are treated in hospital for traumatic brain injury, but there are also hidden statistics to consider [8]. Each year, approximately 1300 people with different tumors in the brain are also diagnosed [9] and finally there are other kind of ABI caused by diseases. For people with ABI, vocational rehabilitation is often a long process [10] and, in Sweden, only 35–41% of them have returned to work after 2–3 years [4, 5]. This is in line with international findings showing that about 40% of individuals with an ABI return to work within 2 years [11]. Thus, the probability of returning to work after an ABI is generally low, which influences both the individual and the society. Several factors have been identified as associated with return to work among people with ABI. In the present study, we have categorized the factors into four areas: person-related factors, injury-related factors, activity-related factors and rehabilitation-related factors.

Regarding the person-related factors age [3, 12, 13] and gender [3, 12, 14–16], older people [3, 12, 13] and women [3, 12, 14–16] have a higher risk of not returning to work after ABI. Education is another important area, in that having a low education level [17–19] increases the risk of not returning to work. For instance, it has been shown that patients without a university degree are 2.3 times less likely to return to work than university graduates [20]. Others have shown that younger patients with stroke who had a university degree were 13% more likely to return to work than those without a university degree [19]. In addition, the individual’s social network is important, for instance being married was found in one study to be a positive predictor for returning to work [21], although others have not found any association between marital status and returning to work [3]. Patients who are less motivated [22, 23], who do not emotionally accept their disability [24], who are inflexible and unrealistic in their vocational goals, or who have a more avoidant coping style [14] are less likely to return to work. Moreover, how patients talk about and understand (or do not understand) their rehabilitation and return-to-work process can influence their opportunities to return to work [25].

In regard to injury-related factors, particularly those related to the degree of injury, such as multiple bodily injuries [16], low physical ability [3, 5, 12, 14, 15, 26–30], and a prolonged stay in hospital [13–15, 17, 24, 31] are significant predictors for taking a longer time to return to work. Moreover, being diagnosed with depression [14, 15, 26], having low cognitive ability [5, 23, 26], and the presence of fatigue [32–34] are all associated with worse return-to-work outcomes.

Concerning activity-related factors, such as performing activities of daily living independently at admission to hospital for a first stroke, were associated with a three times higher chance of returning to work early than for individuals who were totally dependent on others for activities of daily living [3]. One study on cardiac arrest [35] showed that those who were discharged to their own homes had fewer neurological deficits, were more able to handle activities of daily living, had fewer cognitive difficulties, and could more easily return to work. Another relevant factor for an independent lifestyle after ABI was transportation, both for community integration and for vocational rehabilitation [36].

About rehabilitation-related factors, research into individual rehabilitation planning, active participation in inpatient care, and how that affects return to work among patients with ABI is scarce. However, studies have shown that patients with neurological, cardiovascular, and respiratory impairments who were included in the planning of their rehabilitation goals were more compliant with the training regime than those who were not included in the planning [37].

In summary, existing research shows that a variety of factors can affect the opportunities for patients with ABI to return to work. Most research has focused mainly on personal and injury-related factors and less on activity and rehabilitation factors. For instance, there is little research concerning how home support, rehabilitation planning, and possessing a driver’s license affect return to work for patients with ABI. Also, research into person-related factors affecting return to work, such as being born in another country, partnership status, and having children living at home is warranted. In addition, the legal and social framework of a particular country may have consequences for patients with ABI and their return to work. Thus, the aim of this study was to investigate person-related, injury-related, activity-related, and rehabilitation-related risk markers for not returning to work among patients with ABI.

## Method

The regional Ethical Review Board in Uppsala, Sweden (ref. 2016/055) approved the study. After ethical approval, an application was sent to the quality register WebRehab Sweden for data access. The application was agreed, and a statistician from the register extracted data and made it available to the researchers.

### Study Population

**Fig. 1:**
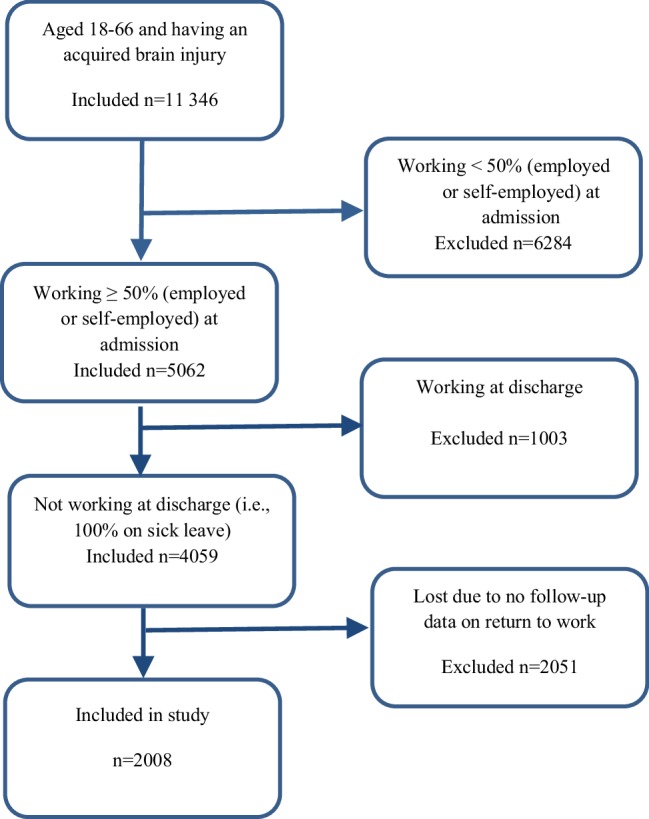
Flow chart of the inclusion process

WebRehab Sweden is a quality register that collects data on brain-injured patients upon admission to hospital, at discharge from hospital, and at follow-up 1 year after the injury [38]. WebRehab Sweden started 1997 and covers 75% of the rehabilitation medicine clinics in Sweden [38]. Between 1 January 2007 and 15 January 2016 (the data collection period for the present study), the register included a total of 11,346 patients with ABI. The inclusion criteria for the present study were: (1) being 18–66 years old (2) having an ABI (i.e., stroke, subarachnoid hemorrhage, traumatic brain injury, post-infectious/post-inflammatory brain injury, anoxic brain injury, or other brain injury), (3) working 50% or more (employed or self-employed) at admission to hospital, (4) not working at all at discharge from hospital (i.e., being on 100% sick leave), and (5) having follow-up data. A total of 2008 patients were included. For a flow chart, see Fig. 1. Those patients who appeared several times in the data file were only included in the study at the first registered injury period. Only those with a maximum hospitalization period of 1 year and a hypothetical chance of returning to work within 1 year were included.

### Measures

#### Dependent Variable (Outcome)

##### Returned to Work

This variable was scored as Yes if the patient worked at least 50%, in paid employment or self-employed, at follow-up 1 year after the injury and No if they were working < 50% at follow-up.

#### Independent Variables (Predictors)

##### Demographic Data

Demographic data consisted of age (in years) at admission, gender, education (compulsory school, upper secondary school, or university), and country of birth (Sweden or outside Sweden). Hospital stay was measured in days from admission to discharge.

##### Diagnosis

Diagnosis was categorized at admission to hospital into seven groups and in this study rescored into three categories: *stroke* (stroke and subarachnoid hemorrhage), *traumatic brain injury*, and *other brain injuries* (post-infectious/post-inflammatory brain injury, anoxic brain injury, brain tumors, and other brain injuries).

##### Functional Outcome

Functional outcome was measured using the Extended Glasgow Outcome Scale (GOSE) [39]. GOSE assesses overall function after a head injury relative to pre-injury function on a global scale in eight steps [40, 41]. The scores range from dead (I), vegetative state (II), lower severe disability (III), upper severe disability (IV), lower moderate disability (V), upper moderate disability (VI), lower good recovery (VII), and upper good recovery (VIII). In this study, the eight GOSE levels were rescored into three categories, with GOSE category I–IV labeled as *poor recovery*, category V–VI as *moderate recovery*, and category VII–VIII as *good recovery*.

##### Motor and Cognitive Function

Motor and cognitive function was measured using the Functional Independence Measure (FIM) [42, 43]. FIM is categorized into 18 activities. Each of the activities is scored from 1 (total need of assistance) to 7 (complete independence). These activities are grouped into six areas of function: (I) self-care, (II) sphincter control, (III) mobility, (IV) locomotion, (V) communication, and (VI) social cognition [42, 43]. FIM is assessed by observing the patient [42]. The activities in category I–IV measure motor function and the activities in category V and VI measure cognitive function. In the present study, the motor function and cognitive function mean scores were rescored into three levels: *total assistance* (a mean score from 1.00 to 2.99), *some help needed* (a mean score from 3.00 to 5.99), and *no help needed* (a mean score from 6.00 to 7.00).

##### Partnership Status and Having Children Living at Home

The register contains seven categories for the household variable, confounding ‘marital status’ and ‘having children’. These variables were rescored into two new variables: Being single (Yes or No) and having children living at home (Yes or No), the alternatives “don’t know” and “other” were excluded from analysis.

##### Home Support

In the register, the housing variable included a mix of accommodation types (ordinary or special) and support in the home (support and no support). This variable was rescored into a new Home support variable scored as those with support (Yes) and those without support (No), regardless of type of accommodation.

##### Driver’s License

Having a driver’s license at discharge was scored as Yes if the patient still had one, and No if the license had been suspended or the patients never had any.

##### Individual Rehabilitation Plan

This tool outlines the intended rehabilitation process for the individual patient. The plan is established jointly with the patient, health care representatives, the municipality, the relatives, and the employer [44]. The aim of the plan is to increase the patient’s participation in his or her rehabilitation process. The WebRehab register contains two questions on this topic: ‘Has a written rehabilitation plan been prepared?’, scored as No or Yes and ‘Has a written rehabilitation plan been used?’, scored as Not used or Yes/partly used.

##### Satisfaction with the Rehabilitation

The register also contains a patient-reported experience measure (PREM) with seven questions regarding the patient’s degree of satisfaction with (1) the attention received from the staff, (2) the cooperation with the staff, (3) the rehabilitation process, (4) the patient’s influence over the rehabilitation process, including the individual rehabilitation plan, (5) the information given about the brain injury, (6) the information given on where to get more support if needed after discharge from hospital, and (7) the information and attention the family and relatives had received during the patient’s rehabilitation at the clinic. Each question was scored on a four-point Likert scale from ‘very dissatisfied’ to ‘very satisfied’. In the present study, the responses ‘very dissatisfied’ and ‘dissatisfied’ were rescored as *dissatisfied* and the responses ‘satisfied’ and ‘very satisfied’ were rescored as *satisfied*. There was also a ‘don’t know’ alternative, which was excluded from the analyses.

##### Health Status

This was measured by five questions covering mobility, self-care, usual activities (e.g. work, studies, household chores, family and leisure activities), pain/discomfort, and anxiety/depression. The questions were taken from the EuroQol five dimensions questionnaire, EQ-5D [45]. Each question was scored in three levels: severe problems, some problems, and no problems. In this study, the questions were treated as individual variables.

### Statistical Analyses

All data analysis was performed using IBM SPSS Statistics version 22.0 (IBM Corp., Armonk, NY, USA). Descriptive statistics (mean and standard deviation) were used to characterize the sample. The risk ratio (RR) and its 95% confidence interval (CI) for returning to work was estimated for each of the predictor variables using the Chi square test with Fisher’s exact test. A p value of 0.05 or less was regarded as statistically significant.

## Results

As shown in Fig. 1, the analyses are based on 2008 patients. Of these, 690 had returned to work and 1318 had not returned to work at follow-up 1 year after the injury. Baseline characteristics of the study sample are shown in Table 1.

**Table 1:** Baseline characteristics at admission of persons who returned to work (RTW; n = 690) or did not return to work (non-RTW; n = 1318) within 357 days after acquired brain injury

Variables	Total N = 2008 (100%)	Non- RTW N = 1318 (66%)	RTW N = 690 (34%)
Person-related factors			
Age group (mean 51.02; SD 10.41)	2008 (100)	1318 (100)	690 (100)
18–29	129 (7)	76 (6)	53 (8)
30–39	181 (9)	112 (9)	69 (10)
40–49	458 (23)	302 (23)	156 (23)
50–59	896 (45)	604 (46)	291 (42)
60–66	345 (17)	224 (17)	121 (18)
Gender	2008 (100)	1318 (100)	690 (100)
Man	1278 (64)	787 (60)	491 (71)
Woman	730 (36)	531 (40)	199 (29)
Country of birth	1248 (100)	785 (100)	463 (100)
Sweden	1099 (88)	667 (85)	432 (93)
Outside Sweden	149 (12)	118 (15)	31 (7)
Education	1180 (100)	743 (100)	437 (100)
Compulsory school (9 years of education)	117 (10)	79 (11)	38 (8)
Upper secondary school (< 12 years of education)	653 (55)	439 (59)	214 (50)
University (> 12 years of education)	410 (35)	225 (30)	185 (42)
Marital status	1918 (100)	1254 (100)	664 (100)
Single	643 (34)	424 (34)	219 (33)
Living with a partner	1275 (66)	830 (66)	445 (67)
Children	1918 (100)	1254 (100)	664 (100)
No children in household	1310 (68)	878 (70)	432 (65)
Children in household	608 (32)	376 (30)	232 (35)
Injury-related factors			
Diagnosis	2008 (100)	1319 (100)	690 (100)
Stroke	1476 (73)	986 (75)	490 (71)
TBI	336 (17)	206 (16)	130 (19)
Other kind of brain injury	196 (10)	126 (9)	70 (10)
Aphasia/dysphasia	1312 (100)	885 (100)	427 (100)
No	981 (75)	630 (71)	351 (82)
Yes	331 (25)	255 (29)	76 (18)
Hospital stay (mean 52.34 days; SD 42.87)	2008 (100)	1317 (100)	690 (100)
Short (0–24 days)	537 (27)	224 (17)	313 (45)
Moderate (25–68 days)	943 (47)	625 (47)	318 (46)
Long (68–357 days)	528 (26)	469 (36)	59 (9)
Functional outcome	1400 (100)	912 (100)	488 (100)
Good recovery (VII–VIII)	308 (22)	127 (14)	181 (37)
Moderate disability (V–VI)	824 (59)	543 (60)	281 (58)
Very severe and severe disability (I–IV)	268 (19)	242 (26)	26 (5)
Motor function	1590 (100)	1073 (100)	518 (100)
No help needed 6–7	1149 (72)	684 (64)	465 (90)
Some help needed 3–5.99	348 (22)	302 (28)	46 (9)
Total assistance 1–2.99	93 (6)	86 (8)	7 (1)
Cognitive function	1591 (100)	1073 (100)	518 (100)
No help needed 6–7	986 (62)	572 (53)	414 (80)
Some help needed 3–5.99	533 (33)	438 (41)	95 (18)
Total assistance 1–2.99	72 (5)	63 (6)	9 (2)
Mobility	1464 (100)	959 (100)	505 (100)
I have no problems walking about	761 (52)	404 (42)	357 (70.5)
I have some problems walking about	663 (45)	517 (54)	146 (29)
I am confined to bed	40 (3)	38 (4)	2 (0.5)
Pain/discomfort	1464 (100)	959 (100)	505 (100)
I have no pain or discomfort	623 (42)	358 (37)	265 (52)
I have moderate pain or discomfort	771 (53)	545 (57)	226 (45)
I have extreme pain or discomfort	70 (5)	56 (6)	14 (3)
Anxiety/depression	1464 (100)	959 (100)	505 (100)
I am not anxious or depressed	809 (55)	468 (49)	341 (67)
I am moderately anxious or depressed	610 (42)	455 (47)	155 (31)
I am extremely anxious or depressed	45 (3)	36 (4)	9 (2)
Activity-related factors			
Self-care	1464 (100)	959 (100)	505 (100)
I have no problems with self-care	1092 (75)	640 (67)	452 (89)
I have some problems washing or dressing myself	327 (22)	278 (29)	49 (10)
I am unable to wash or dress myself	45 (3)	41 (4)	4 (1)
Usual activities	1464 (100)	959 (100)	505 (100)
I have no problems with performing my usual activities	459 (31)	231 (24)	228 (45)
I have some problems with performing my usual activities	709 (49)	493 (51)	216 (43)
I am unable to perform my usual activities	296 (20)	235 (25)	61 (12)
Driver’s license	1909 (100)	1250 (100)	659 (100)
No	1762 (93)	1167 (93)	595 (90)
Yes	147 (7)	83 (7)	64 (10)
Home support	259 (100)	152 (100)	107 (100)
Accommodation without support	244 (94)	140 (92)	104 (97)
Accommodation with support	15 (6)	12 (8)	3 (3)
Rehabilitation-related factors			
The rehabilitation process	1427 (100)	932 (100)	495 (100)
Dissatisfied	16 (1)	16 (2)	0 (0)
Satisfied	1411 (99)	916 (98)	495 (100)
The individual’s cooperation with the staff	897 (100)	2 (100)	895 (100)
Dissatisfied	556 (62)	2 (100)	554 (62)
Satisfied	341 (38)	0 (0)	341 (38)
The individual’s influence over the rehabilitation process including his or her rehabilitation plan	857 (100)	533 (100)	324 (100)
Dissatisfied	27 (3)	24 (4)	3 (1)
Satisfied	830 (97)	509 (96)	321 (99)
The information given about the brain injury	1411 (100)	911 (100)	499 (100)
Dissatisfied	85 (6)	63 (7)	22 (4)
Satisfied	1325 (94)	848 (93)	477 (96)
The information on where to get support if needed after discharge from hospital	1268 (100)	821 (100)	447 (100)
Dissatisfied	66 (5)	49 (6)	17 (4)
Satisfied	1202 (95)	772 (94)	430 (96)
The attention given to the individual by the staff	1446 (100)	942 (100)	505 (100)
Dissatisfied	11 (1)	10 (1)	1 (1)
Satisfied	1435 (99)	931 (99)	504 (99)
Has a written rehabilitation plan been prepared?	2008 (100)	1318 (100)	690 (100)
No	214 (11)	116 (9)	98 (14)
Yes	1794 (89)	1202 (91)	592 (86)
Has a written rehabilitation plan been used?	1799 (100)	1205 (100)	594 (100)
No	12 (0.5)	5 (0.5)	7 (1)
Yes	1787 (99.5)	1200 (99.5)	587 (99)
The information and attention the family and relatives had received during the individual’s rehabilitation at the clinic	828 (100)	514 (100)	314 (100)
Dissatisfied	19 (2)	13 (2)	6 (2)
Satisfied	809 (98)	501 (98)	308 (98)

The risk ratios for not returning to work are given in Table 2. The results showed that, among the person-related factors, being a woman, being born outside Sweden, having a lower educational level, and not having children in the household increased the risk of not returning to work. Among the brain injury-related factors, a hospital stay of more than 25 days increased the risk of not returning to work. In addition, those with aphasia had a larger risk of not returning to work than those without aphasia. Concerning motor and cognitive functions measured by the FIM, those classified as needing *total assistance* or *some help needed* had a higher risk of not returning to work than those who did not need help. Both those with moderate disability and those with poor recovery according to the GOSE measure had a greater risk of not returning to work than those with good recovery. In addition, those who were confined to bed or had problems with mobility were less likely to return to work. Furthermore, patients who had pain/discomfort or anxiety/depression had a higher risk of not returning to work compared to those with no pain/discomfort or anxiety/depression problems. There was no significant difference in the risk of not returning to work in relation to the type of brain injury diagnosis.

**Table 2:** Relative risk (RR) of not returning to work after acquired brain injury

Variables	RR of not returning to work	95% confidence interval	P^a^
Person-related factors			
Age group			
18–29	Ref	Ref	
30–39	1.05	0.87–1.28	0.638
40–49	1.07	0.98–1.18	0.146
50–59	1.05	1.00–1.11	0.058
60–66	1.07	0.95–1.21	0.240
Gender			
Man	Ref	Ref	
Woman	1.40	1.22–1.60	< 0.001
Country of birth			
Sweden	Ref	Ref	
Outside Sweden	2.25	1.54–3.28	< 0.001
Education			
Compulsory school (9 years)	Ref	Ref	
Upper secondary school (≤ 12 years)	1.00	0.94–1.06	1.000
University (> 12 years)	0.89	0.82–0.98	0.015
Marital status			
Single	Ref	Ref	
Partnership	0.99	0.93–1.06	0.722
Children			
No children in household	Ref	Ref	
Children in household	0.86	0.75–0.98	0.030
Injury-related factors			
Diagnosis			
Stroke	Ref	Ref	
TBI	0.82	0.68–1.00	0.056
Other kind of brain injury	0.91	0.69–1.19	0.519
Aphasia/dysphasia			
No	Ref	Ref	
Yes	1.62	1.29–2.04	< 0.001
Hospital stay			
Short (0–24.99 days)	Ref	Ref	
Moderate (25–68.99 days)	1.46	1.34–1.59	< 0.001
Long (69–357 days)	4.27	3.36–5.42	< 0.001
Functional outcome			
Good recovery VII–VIII	Ref	Ref	
Moderate disability V–VI	1.33	1.23–1.45	< 0.001
Very severe and severe disability I–IV	5.22	3.62–7.54	< 0.001
Motor function			
No help needed (6.00–7.00)	Ref	Ref	
Some help needed (3.00–5.99)	3.40	2.54–4.55	< 0.001
Total assistance (1.00–2.99)	7.53	3.52–16.13	< 0.001
Cognitive function			
No help needed (6.00–7.00)	Ref	Ref	
Some help needed (3.00–5.99)	2.32	1.91–2.82	< 0.001
Total assistance (1.00–2.99)	4.66	2.35–9.27	< 0.001
Mobility			
I have no problems walking about	Ref	Ref	
I have some problems walking about	1.93	1.67–2.24	< 0.001
I am confined to bed	15.43	3.75–63.53	< 0.001
Pain/discomfort			
I have no pain or discomfort	Ref	Ref	
I have moderate pain or discomfort	1.31	1.18–1.46	< 0.001
I have extreme pain or discomfort	2.70	1.53–4.75	< 0.001
Anxiety/depression			
I am not anxious or depressed	Ref	Ref	
I am moderately anxious or depressed	1.58	1.36–1.83	< 0.001
I am extremely anxious or depressed	2.78	1.36–5.69	0.003
Activity-related factors			
Self-Care			
I have no problems with self-care	Ref	Ref	
I have some problems washing or dressing myself	3.10	2.33–4.11	< 0.001
I am unable to wash or dress myself	6.86	2.48–19.03	< 0.001
Usual activities			
I have no problems with performing my usual activities	Ref	Ref	
I have some problems with performing my usual activities	1.40	1.26–1.56	< 0.001
I am unable to perform my usual activities	2.39	1.88–3.04	< 0.001
Driver’s license			
No	Ref	Ref	
Yes	0.68	0.50–0.93	0.019
Home support			
Accommodation without support	Ref	Ref	
Accommodation with support	2.82	0.81–9.74	0.107
Rehabilitation-related factors			
The rehabilitation process			
Dissatisfied	Ref	Ref	
Satisfied	0.98	0.98–0.99	0.002
The individual’s cooperation with the staff			
Dissatisfied	Ref	Ref	
Satisfied	1.00	0.99–1.00	0.528
The individual’s influence over the rehabilitation process including his or her rehabilitation plan			
Dissatisfied	Ref	Ref	
Satisfied	0.96	0.94–0.99	0.004
The information given about the brain injury			
Dissatisfied	Ref	Ref	
Satisfied	0.97	0.95–1.00	0.062
The information on where to turn to get support if needed after discharge from hospital			
Dissatisfied	Ref	Ref	
Satisfied	0.98	0.95–1.00	0.112
The attention given to the individual by the staff			
Dissatisfied	Ref	Ref	
Satisfied	0.99	0.98–1.00	0.109
Has a written rehabilitation plan been prepared?			
No	Ref	Ref	
Yes	1.06	1.03–1.10	< 0.001
Has a written rehabilitation plan been used?			
No	Ref	Ref	
Yes	1.01	1.00–1.02	0.071
The information and attention the family and relatives had received during the individual’s rehabilitation at the clinic			
Dissatisfied	Ref	Ref	
Satisfied	0.99	0.97–1.02	0.639

^a^Chi-square test with Fischer’s exact test

Concerning the activity-related factors, patients with low ability to perform self-care or usual everyday activities had a higher risk of not returning to work than those who had no problems with these activities. Having one’s driver’s license suspended at discharge also increased the risk of not returning to work. The presence or absence of home support was not a significant risk marker for not returning to work.

Concerning rehabilitation-related factors, the patients’ satisfaction with the information they received about the brain injury, information to the family, information on where to turn with questions related to the brain injury after discharge from the hospital, and the cooperation with the staff, were unrelated to the risk of not returning to work. However, being satisfied with the rehabilitation process and being satisfied with their own influence over the rehabilitation planning process were associated with a higher likelihood of returning to work. Contrary to expectations, having a rehabilitation plan increased the risk of not returning to work. Finally, neither the patients’ satisfaction with the attention from staff or whether an existing written rehabilitation plan had actually been used were significantly related to the risk of not returning to work.

## Discussion

The purpose of this study was to investigate risk markers for not returning to work among patients with ABI. The main result showed that several factors influence the risk of not returning to work. In keeping with previous research, women had a greater risk of not returning to work within a year after a brain injury compared to men [3, 12, 14–16]. In Sweden, almost as many women as men participate in the workforce [46]. Nevertheless, women in Sweden may still be discriminated in working life; for example, gender norms that exist within a specific workplace could make returning to work harder for women with ABI [47].

The results showed that individuals born outside Sweden had higher risk of not returning to work than those born in Sweden. Statistics Sweden report that people born in Sweden have a working rate of 84% while people born outside Sweden have a rate of 71% [48]. No studies about immigrants with ABI, and return to work were found, but a study on low back pain compared native-born patients and immigrants entering rehabilitation noted that immigrants might have special needs that should be addressed when planning rehabilitation [49] which also may be the case for patients with ABI.

The results showed that a high education level was essential for returning to work. Having a university degree increased the chances of returning to work after an ABI, something that has also has been found by others [17–19]. An explanation is that a university degree gives a wider range of possible job assignments. In our study, 45% of the individuals with a university degree returned to work, but only 32% of those with compulsory school education returned to work. A higher education level generally predicts higher income and higher socioeconomic status [19]. Higher education is also often associated with white-collar jobs, and these positions are often more flexible [5, 24, 50]. Therefore, giving the patients the possibility of education during the rehabilitation period could be a positive factor for at least some of these patients, but the education would have to be adapted to the patient’s individual capacity.

Those in households with children had a significantly higher likelihood of returning to work than those in households without children. No other research findings have been found in this area, but children in the household can be a driving force [51]. Returning to working life makes it possible to show the children that everything is normal again after the injury. Financial needs may also be a driving force for return to work.

Moreover, it has been argued that living in a relationship provides a natural support system for an individual with brain injury [21], but, in our study, being married or living with a partner was not associated with a higher likelihood of returning to work, and similar results has been found by other studies [3, 52]. Nevertheless, there are some findings that indicate that marriage may be a positive predictor for returning to work [21] and others have shown that support from family is important for patients with brain injuries on returning to work [53, 54].

The present study did not find age to be a risk marker for not returning to work. This is contrary to previous findings, that younger stroke patients could more easily return to work because they had less indicators of stroke severity, such as hypertension or diabetes prior to their stroke [12].

In regard to injury-related factors, patients with lower motor function showed a higher risk of not returning to work, which is in line with findings in many other studies [3, 5, 12, 14, 15, 26–30]. Those who needed some help with cognitive function (FIM) and those who needed total assistance had a higher risk of not returning to work. The patients who had problems with cognition, continually needed cognitive support, and required help at work had a more complex return-to-work process, as shown in several other studies [5, 23, 26].

A long period of hospital stay was another risk marker for not returning to work. Those with a longer stay in hospital had a many times higher risk of not returning to work than those with a shorter hospital stay. Several other studies have found a similar result [13–15, 17, 24, 31]. Some of them explained that the longer stay in hospital was found with patients who had a more severe injury, making it harder to return to work [15, 17, 24, 31].

Patients with aphasia had a higher risk of not returning to work than those without aphasia. A review showed that employment decreased after aphasia and return to work was often at a less demanding level [55]. Patients with aphasia experienced that it was a struggle to handle activities of daily living and that their aphasia had an impact on their participation in society [56].

Those with moderate or extreme pain/discomfort had higher risk of not returning to work than those without pain/discomfort. This is in line with previous research, which showed that severe head and/or bodily pain after mild traumatic brain injury predicted a delayed return to work [57]; this is possibly also the case for patients with other kinds of ABI. Finally, our results showed that it was harder to return to work if the patient also suffered from anxiety or depression, which is in keeping with other studies [14, 15, 26].

There was, however, no increased risk of not returning to work in relation to type of diagnosis, indicating that the diagnosis may play a lesser role than the individual’s functional level.

Those with poor self-care ability in activities of daily living, such as dressing and washing themselves, had an increased risk of not returning to work. This result is consistent with other studies showing that patients who can independently perform activities of daily living return to work earlier after the injury [3]. In our study, those who had problems with their usual activities, such as studies, household chores, and family and leisure activities, had a higher risk of not returning to work. In addition, those who had their driver’s license suspended had a higher risk of not returning to work, which is in line with other findings that having a driver’s license was associated with a productive lifestyle after ABI [58]. However, having home support did not influence the risk of not returning to work.

Finally, regarding rehabilitation-related factors satisfaction with the rehabilitation process and the individual’s influence over the process, including the individual rehabilitation plan, increased the chances of returning to work. This is in line with other research showing that patients who could understand the rehabilitation were more likely to return to work [25].

One way to help patients understand the rehabilitation is to form a rehabilitation plan that create a predictable vocational rehabilitation process that is transparent for the patient and the staff. However, our results showed, counterintuitively, that having a written rehabilitation plan was associated with a greater risk of not returning to work, regardless of whether this written individual rehabilitation plan was used or not. It may be that patients with minor injuries and a short rehabilitation period are less likely to get a rehabilitation plan and more likely to be able to return to work without needing such support. Another possible explanation is that patients with brain injury have rehabilitation plans from different hospital departments and other organizations that are not coordinated, so the plans that were created have not actually been used or followed up. Future research may shed some light on this result.

Although previous research has shown that information given to the patient and family is an important aspect in the return-to-work process [23], our study found no association between satisfaction with information about the brain injury, satisfaction with information on where to turn for information after the hospitalization period, or satisfaction with the information and attention given to the family and returning to work.

### Study Limitations

Although there are strengths with the study regarding sample size, there are some limitations that have to be addressed, such as the relatively large proportion of missing data. The lack of data is mostly due to the fact that many of the variables were optional for the clinics to assess. As a result, only 18% of the total cohort could be included in the present study. Another limitation is that there were no data on the exact number of days between discharge from hospital and the time when the patient returned to work, or on what percentage of full-time work they started with and at what point they increased their working hours. Finally, a weakness is that the quality register provides fixed variables, which limits the type of possible research questions [59]. Consequently, in our study, it would have been interesting to examine more rehabilitation-related risk markers as well as risk markers related to adjustments in the vocational rehabilitation process.

## Conclusion

The present study showed that all areas of person-related, injury-related, activity-related, and rehabilitation-related factors are associated with the likelihood of returning to work for patients with ABI. The return-to-work process is complex, with all areas interacting with each other to increase the risk of not returning to work. Most notably, being a woman, being born outside of Sweden, having only a compulsory school diploma, and not having children in the household increased the risk of not returning to work after ABI. Of the injury-related factors, long hospital stay, aphasia, low motor function, low cognitive function, high pain/ discomfort, and high anxiety/depression worsened the chances of returning to work. Of the activity-related factors, low function in self-care, low function in usual activities, and low personal influence over the rehabilitation plan also gave a higher risk of not returning to work after ABI. The results show that several factors facilitate return to work, which support previous research proposing individualized work rehabilitation for returning to work after an ABI. Clinical implications for rehabilitation practitioners are therefore to involve patients in individual planning and *follow-up on vocational rehabilitation and return to work outcomes*.
